# Deep learning-based user experience evaluation in distance learning

**DOI:** 10.1007/s10586-022-03918-3

**Published:** 2023-01-08

**Authors:** Rahim Sadigov, Elif Yıldırım, Büşra Kocaçınar, Fatma Patlar Akbulut, Cagatay Catal

**Affiliations:** 1 Department of Computer Engineering, Istanbul Kültür University, Istanbul, Turkey; 2 Department of Software Engineering, Istanbul Kültür University, Istanbul, Turkey; 3 Department of Computer Science and Engineering, Qatar University, Doha, Qatar

**Keywords:** Distance learning, Sentiment analysis, Deep learning, NLP

## Abstract

The Covid-19 pandemic caused uncertainties in many different organizations, institutions gained experience in remote working and showed that high-quality distance education is a crucial component in higher education. The main concern in higher education is the impact of distance education on the quality of learning during such a pandemic. Although this type of education may be considered effective and beneficial at first glance, its effectiveness highly depends on a variety of factors such as the availability of online resources and individuals’ financial situations. In this study, the effectiveness of e-learning during the Covid-19 pandemic is evaluated using posted tweets, sentiment analysis, and topic modeling techniques. More than 160,000 tweets, addressing conditions related to the major change in the education system, were gathered from Twitter social network and deep learning-based sentiment analysis models and topic models based on latent dirichlet allocation (LDA) algorithm were developed and analyzed. Long short term memory-based sentiment analysis model using word2vec embedding was used to evaluate the opinions of Twitter users during distance education and also, a topic model using the LDA algorithm was built to identify the discussed topics in Twitter. The conducted experiments demonstrate the proposed model achieved an overall accuracy of 76%. Our findings also reveal that the Covid-19 pandemic has negative effects on individuals 54.5% of tweets were associated with negative emotions whereas this was relatively low on emotion reports in the YouGov survey and gender-rescaled emotion scores on Twitter. In parallel, we discuss the impact of the pandemic on education and how users’ emotions altered due to the catastrophic changes allied to the education system based on the proposed machine learning-based models.

## Introduction

The Covid-19 pandemic has made it necessary to make changes in our social life to protect ourselves against the virus [1] while continuing our daily activities. One of the most affected areas by the pandemic is education. Due to the adverse conditions that occurred during the pandemic, face-to-face education was suspended by governments worldwide and online education took its place for a while. Such an unfamiliar situation at a very large scale led to different conflicting ideas among people. Some thought this new system would have a positive effect on students, some criticized it and pointed out the flaws of distance education. Because of social isolation during the pandemic, people mostly shared their opinions on digital platforms. Twitter was still very popular during the pandemic period. The fact that every tweet has different kinds of content such as restrictions, slang, hyperlinks, emojis, and hashtags make Twitter sentiment analysis a challenging process [2]. Considering such complexities, Natural Language Processing (NLP) techniques are very beneficial while building different solutions [3].

These days, one of the most popular research areas of NLP is sentiment analysis [4, 5], which is employed in diverse domains such as recommender systems, data-driven systems, healthcare studies, etc. Due to the popularity of social media, enormous amounts of data are being created daily on the Internet. Individuals share their opinions on various social media platforms such as Facebook, YouTube, and Twitter, and as such, it is possible to reach interesting and useful conclusions and also develop products by analyzing sentiments from these posts. Because these reviews not only produce an impact on the person’s perspective but also provoke an effect on product deals [6] that have an effect on third-party dealers on retail platforms [7]. However, constructing such predictions is not an easy task, since the oversaturation of the content fills the channels with too much noise, making it difficult to connect and convert. It makes a robust sentiment analysis very challenging with this kind of unfiltered content that includes grammar mistakes, typos, links, and emojis.

The main motivations of this study are to analyze the sentiments of the tweets using Deep Learning models, see how the education system was affected during the Covid-19 epidemic, and how distance education affected people socially. The objective is to help the management groups who design the education systems and individuals accurately map the emotions during the pandemic by analyzing the emotions of individuals on distance education through social media content. In this study, sentiment analysis was carried out using a dataset collected from the Twitter social media platform. The emotion class of each text was determined and the polarity values were calculated. We gathered Turkish tweets about distance education. The study contains several procedures such as pre-processing, tweet labeling according to the polarity values, categorizing data by their content, training model with LSTM, Recurrent Neural Networks (RNN) algorithms, and optimization of models by tuning the hyperparameters. The main contributions of this study are as follows:

- A new dataset was built for topic-oriented sentiment analysis in the Turkish language
- Efficient deep learning-based sentiment classification models were developed using different deep learning and word embedding algorithms
- Social media was used to critically evaluate the education system, which is one of the most sensitive and important matters of today, especially during a pandemic.

## Background and related work

### Natural language processing and sentiment analysis

Sentiment analysis is a method of scientific inference, which is very useful for the daily life of people on a global scale, especially on social media, where people share their opinions on different issues such as products, services, institutions, and brands, especially social and political developments. It is the act of fast and reliable reporting and making meaning from large volumes of data on various online platforms [8]. It is one of the most important problems in NLP today. The field of NLP is the whole of methods formed by the interactive work of machine learning and computational linguistics fields with computer science [9]. In addition to many application areas, NLP also deals with the digitalization of texts with the help of software programs. Individuals share the information they have on social media platforms, their thoughts on different subjects, and make comments about the movies they watch, the books they read, and the products they buy [10]. Such data shared on social media platforms is enormous and therefore, the opportunity to measure the polarity of this data is of great interest to experts and data scientists.

The purpose of sentiment analysis is to measure the polarity and calculate the sentiment polarity values of the texts. Therefore, sentiment analysis can be considered as a text classification method. Each tweet in the dataset included in the sentiment analysis studies categorizes the content on a broader scale such as positive, negative, and neutral [11]. It is basically a text-processing action and aims to determine what the given text expresses emotionally. Based on this, sentiment analysis can be expressed as the process of categorizing and classifying the predominant emotion in the written message. On the other hand, sentiment classification is basically formed by the use of opinion mining techniques and some statistical techniques [12].

Recently, deep learning-based analysis of sentiments has achieved great success in different areas such as audio-visual [13], image [14], and sequential data [15] processing. While increasing the training data for traditional machine learning techniques does not always improve the performance, in deep learning, the success chance increases as the training data diversifies and increases. In this case, a large amount of training data is required [16]. There are many proven deep learning algorithms for sequential data processing such as Recurrent Neural Networks (RNN), LSTM, 1D-Convolutional Neural Networks (CNN), and Gated Recurrent Units (GRU). For instance, Imran et al. [17] used an LSTM architecture to analyze the correlation between people’s sentiments and emotions with a dataset that was gathered during the Covid-19 pandemic. Moreover, sentiment analysis is performed with hybrid models that combine different deep learning architectures [18–20].

Despite remarkable progress in the evolution of common NLP applications, context-dependent mining, and NLP services, little progress has been made in enhancing existing text analysis capabilities. In the existing design, it is challenging to incorporate an NLP tool with the existing applications. To overcome the issue, several researchers [21, 22] have implemented service-oriented architectures which is currently the most preferred approach in developing this software that is positioned under AI such as NLP, defines a way to make software components reusable and interoperable through service interfaces [23]. Generally, the most important benefits of using the SOA concept are technology reuse and change agility, which cannot be offered in monolith structures. DevOps (Development & Operations) [24], used in developing SOA-based components, is a set of practices for developing, testing, and deploying software quickly and reliably, ensuring harmony between different roles in software teams, and encouraging collaboration among developers, testers, and operators. SOA along with DevOps practices gives rise to Microservices [25, 26], making machine learning models easier to enhance. This highlights the MLOps (Machine Learning Operations) concept that traditional aspects of service monitoring apply differently [27]. AI models can be easily integrated into software systems, as dealing with changing requirements for data, feature modeling, monitoring, debugging, and updating models can be easily performed with MLOps. Studies in the literature present model architectures for solving problems [28–30], as well as explain how these models are integrated into software systems using MLOps.

### Topic modelling

Topic modeling helps arrange a huge amount of textual data by dividing them by the number of different topics found in the text. The method summarizes the text similar to the sentiment analysis approach, however, the difference is that the concern of topic modeling is the topic itself while sentiment analysis is based on the polarity of the given data. The findings derive that, with topic modeling, a model discovers hidden patterns between different topics, helps create annotations according to the sub-genres, and shows branches that simplify the process of summarizing the text [31]. The method considerably inhibits the occurrence of failures in the summarizing part since it is successful for learning if trained in the right way [32], it interactively evaluates the homogeneous data found and then, builds the topics on top of that if a number of topics desired are not given. An article [33] highlights the importance of clustering the words for aircraft accidents and implies that text mining approaches such as topic modeling can play a crucial role in understanding complex systems where both qualitative and quantitative data should be evaluated.

## Methodology

**Fig. 1:**
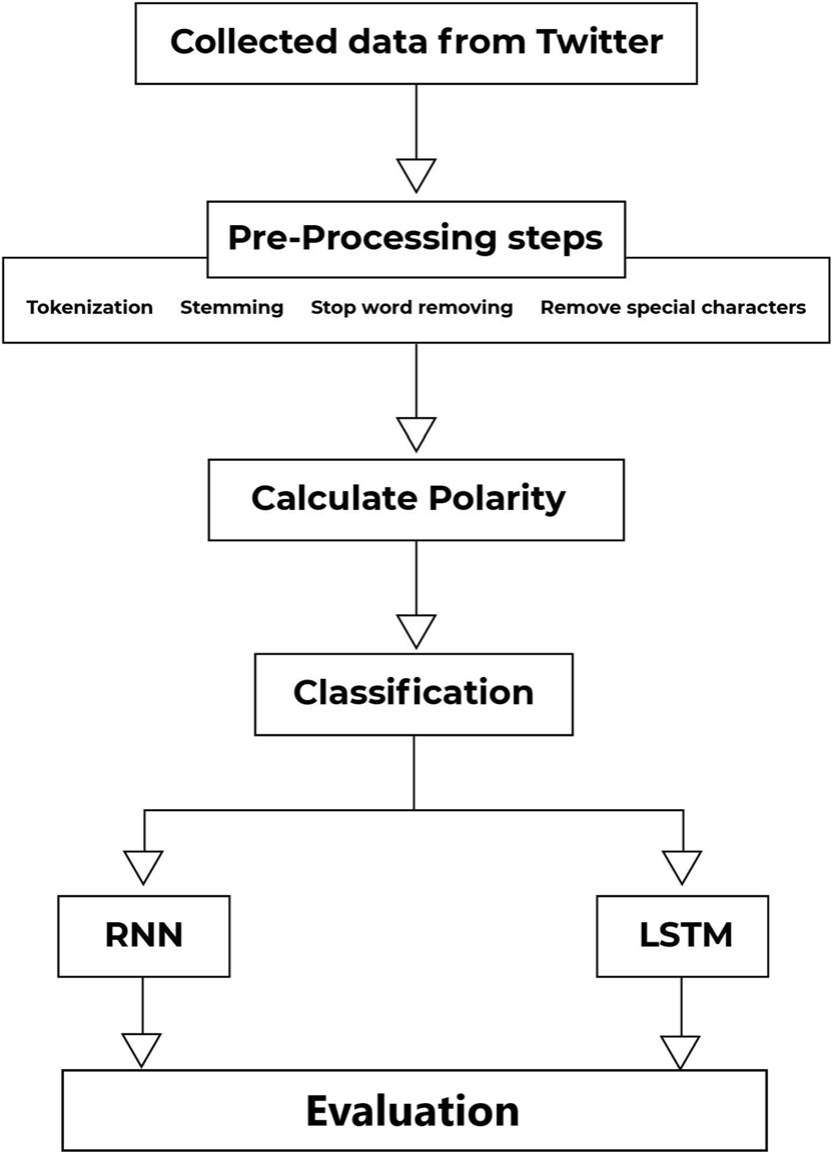
Information flow logic of the proposed system

The current study explores the effect of Covid-19 on Twitter from the perspective of emotion and topics by utilizing pre-trained models, topic modeling, and deep learning-based sentiment analysis models. The proposed system architecture used in this study is shown in Fig. 1.

Data collection is carried out according to the keywords determined in the first step of the study. Pre-processing steps such as tokenization, stemming, removing stop words, and special characters are also applied. In the next step, polarity is calculated and a positive or negative tag is assigned for each tweet. At the end of the classification process, results and learning processes for various model architectures are evaluated.

### Tweet dataset

**Fig. 2:**
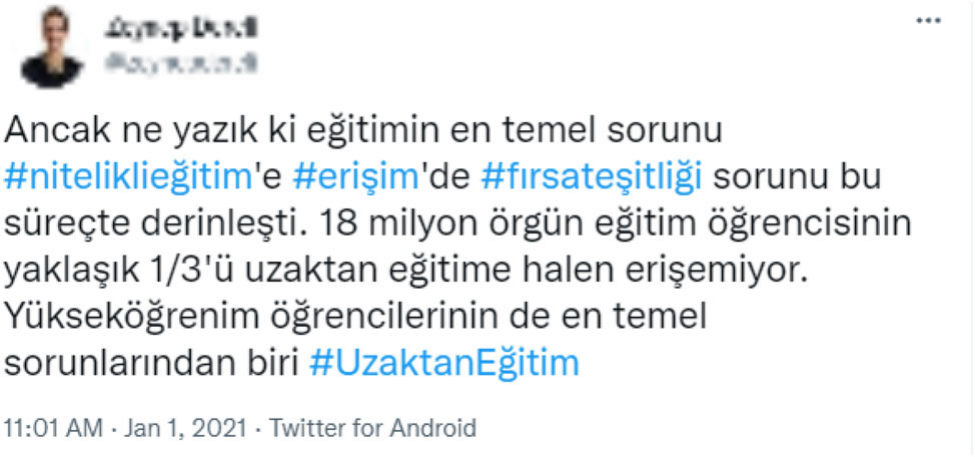
Tweet sample regarding distance education

In the study, we scraped tweets from Twitter with an open-source scraping tool Twint^1^. While building our corpus, we used the following keywords in Turkish: “distance education”, “education”, “distance learning”, “e-learning”, “online education”. We gathered a total of 160,000 tweets^2^, and the timeline of the tweets is between 2020 and 2021. Figure 2 shows one sample from the collected tweets.

### Tweet pre-processing

Tweets often consist of incomplete phrases, various noise, and poorly structured sentences due to the presence of abbreviations, incorrect grammar, malformed words, and non-dictionary terms. For these reasons, it is essential to make the raw text ready for mining. Since almost every tweet contains unnecessary content such as punctuation, hashtags, symbols, and emoticons, they have to be filtered out. The dataset prepared in this research was obtained by applying various pre-processing techniques. The most basic process applied is cleaning the noisy data by removing the expressions that are unnecessary and have no value in sentiment analysis. With the help of this method, various symbols such as links starting with “http” in the content of the texts, emojis, numeric expressions, punctuation marks, and usernames (starting with the @ sign) in the texts belonging to Twitter have been removed. After these processes, all the content of the texts was converted to lowercase letters, and the spaces at the beginning and end of the texts were removed.

After noisy data cleaning on texts, the next data cleaning step is to remove the stop words in Turkish (e.g., acaba, da, her, gibi in Turkish language) that usually represent the most common words in a language. The reason why these words were removed is that they create some misunderstandings or do not have any significance. Later, we applied stemming/lemmatization approach to Turkish text collection. This process was carried out using Zemberek [34], which is an NLP library developed for simplifying words into their roots in the Turkish language. In this way, we were able to find the roots of words. The other feature that we use in Zemberek is to check the spelling. In Table 1, we show some examples of spell-checking and word rooting with Zemberek.

**Table 1:** Tweet samples before and after pre-processing

Raw tweet	After preprocess
Uzzaktan eğitim derslerimizde hocalarımız bizler icin kolaylıklar saglamak adına anlatdıkları video kayıtlar halinde bize ilettiyor	Uzaktan eğitim ders hoca biz kolay sağla anlat video kayıt biz ilet
Yahu insan gunde bilgisayar karsısında oturur, uzaktan egitim cooook kotu	insan gün bilgisayar karşı oturmak uzaktan eğitim çok kütü
Uzaktan egitimin tadini cikaralim kisa zamanda egitim eski haline doner	uzaktan eğitim tat çıkarmak kısa zaman eğitim eski hal dönmek

### Word embedding

Successful text representation starts with a vector space model for representing text, which has been mainly employed for document representation and lately, it has been extended to a word or term representation. We used word embeddings to build neural language models based on word vectors by the Word2Vec toolkit [35]. Word embeddings are reflections in a word space that are expected to preserve semantic and syntactic resemblance between them [36]. It has two architectures to represent words in a multi-dimensional space: the skip-gram (SG) and the continuous bag-of-words (CBOW) model. The CBOW model targets learning the embeddings by predicting the central word in a text and showing the other words without respect to their order. The SG model uses the contrary methodology of CBOW to indicate the encompassing context words given the central word. In the Word2Vec model, skip-gram architecture is used to extract concept categorization by vectorizing words. All cleaned training data is used to create word embeddings and the vector size was specified as 50. The minimum word frequency is 5, which means that the words occurring less than 5 times are not placed in the Word2Vec model. Window, the maximum distance between the current and predicted word in a sentence, is 5. Workers, which are threads for training the model, were selected 4 for faster training.

### Sentence level sentiment polarity calculation

The next step is to determine the polarity score of the tweets, which is a lexicon-based sentiment feature. Since we have a limited dataset in a narrow scope, we fine-tuned tweet content on a new dataset and used the Turkish Electra pre-trained model^3^ to predict the sentiments as positive or negative. The following steps were followed to determine the emotion classes of the tweets.

1. The words that makeup all the texts in the dataset are considered as independent words.
2. Afterwards, these words were checked from two separate vocabularies containing positive and negative words created for the Turkish language.
  If the number of positive words in the text is higher than the number of negative words, this text is labeled as positive,
  otherwise, it is labeled as negative.

As a result of this, we classified the tweets into two categories: negative and positive. We dropped neutral tweets as it is mentioned above. As seen in the Table 2, at the end of this step, a total of 141,438 tweets were classified as 64,332 negatives and 77,106 positive tweets.

**Table 2:** Dataset sentiment class

Emotion	Count	Percentage (%)
Positive	64332	45.5
Negative	77106	54.5
Total	141,438	100

The score of each word is computed by measuring the Pointwise Mutual information (PMI) between two words, $w_{1}$ and $w_{2}$ of the tweet [37]:

1 $$\begin{aligned} PMI(w_{1},w_{2}) = log_{2}(p(w_{1} and w_{2})/p(w_{1})p(w_{2})) \end{aligned}$$

where $p(w_{1} and w_{2})$ is the likelihood of how often $w_{1}$ and $w_{2}$ are repeated, $p(w_{1})$ is likelihood of occurrence of $w_{1}$ and $p(w_{2})$ is likelihood of event of $w_{2}$. Then, for words *w* in a tweet, a sentiment score is computed:

2 $$\begin{aligned} SC(w)= PMI(w, ps) - PMI(w, ng) \end{aligned}$$

where w is a term in the dictionary, *PMI*(*w*, *ps*) is the PMI score between the expression *w* and the positive *ps* content, and *PMI*(*w*, *ng*) is the PMI score between the term *w* and the negative *ng* tweet content. Accordingly, a negative *SC*(*w*) denotes that there is a more powerful relationship between the term *w* with negative emotion and the other way around.

3 $$\begin{aligned} PMI(w, ps)= \sum _{l \in words} PMI(w, l) \end{aligned}$$

If $SC(w) > 0$, then w is positive, otherwise, it is negative.

After all these steps specified above, the next step is text vectorization. Before this stage, the dataset needs to be splitted into two sets, namely training and testing. 20% of the dataset was used for testing and the remaining 80% was used for training. After splitting the dataset as training and testing, we performed text vectorization. The purpose of this process is to translate data written in human language into a language that a deep learning model can understand.

### Sentiment analysis using LSTM and Word2Vec embeddings

**Fig. 3:**
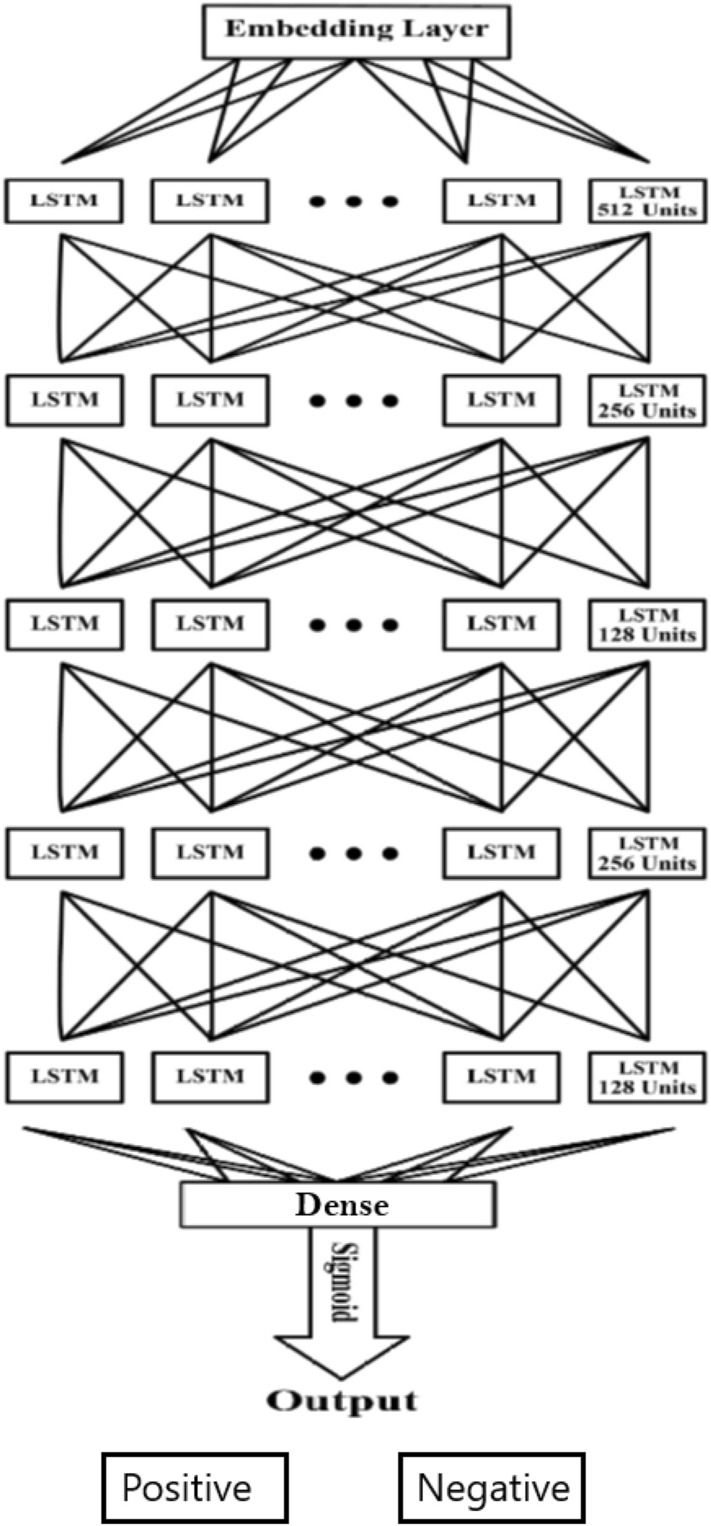
Illustration of proposed LSTM model for Twitter sentiment analysis

The proposed solution for constructing the model first converts the texts into numerical data. Afterward, pre-processing techniques are applied to the input data. The data is then transmitted to the multiple repeating layers for processing. The data is transmitted to the layer where an activation function is running for classification. In Fig. 3, we see the architecture of our LSTM model. An embedding layer, three LSTM layers, and one output layer using the sigmoid function as an activation function are added to the LSTM model that has 512, 256, 128, 256, and 128 neurons with a dropout of 10 percent. The model was compiled with the Adam optimizer using a 0.00001 learning rate and trained in 10 epochs.

### Latent dirichlet allocation-based topic modeling

The text structure is viewed as a mix of topics that can be generated with machine learning methods to predict latent variables. Therefore, it is likely to use multiple methods to obtain information from a sequence of text, i.e., $X= {X_1, X_2, X_3,\ldots , X_n}$ where $X_i$ refers to the $i^{th}$ tweet. LDA is one of the most widely used methods that is characterized as a generative probabilistic model of a sequence. It is a topic modeling method that focuses on probabilities. Let $Z={z_1, z_2,\ldots , z_k}$ be the set of k topic models. We can define a log-likelihood objective function as [38]:

4 $$\begin{aligned} log P(X|Z) = \sum _{x\in X} log p(x|z_\theta (x)) \end{aligned}$$

where $\theta (x)= argmax log p(x|z_\theta )$ is the topic identity of tweet. A topic was documented as a likelihood assembly procedure over all the corpus that has a probability ranging from 0 to 1. In the LDA generator process, the document is treated as a bag of words that is a mixture of topics that contain a mixture of words [39].

For the evaluation of positive and negative topic models, we used perplexity and topic coherence, which deliver a suitable criterion to consider how promising a topic model is. When we achieve lower perplexity, it means that it is a useful model. With topic coherence, we measured the semantic similarity of the group of content. The more coherent the subject is, the higher the average pairwise similarity between words is.

## Experimental results

After different pre-processing steps, approximately 141,438 tweets containing positive or negative emotions remained in the dataset. For a complete analysis, we first carried out the task of topic modeling and then, classified the tweet sentiment analysis using deep learning algorithms.

**Fig. 4:**
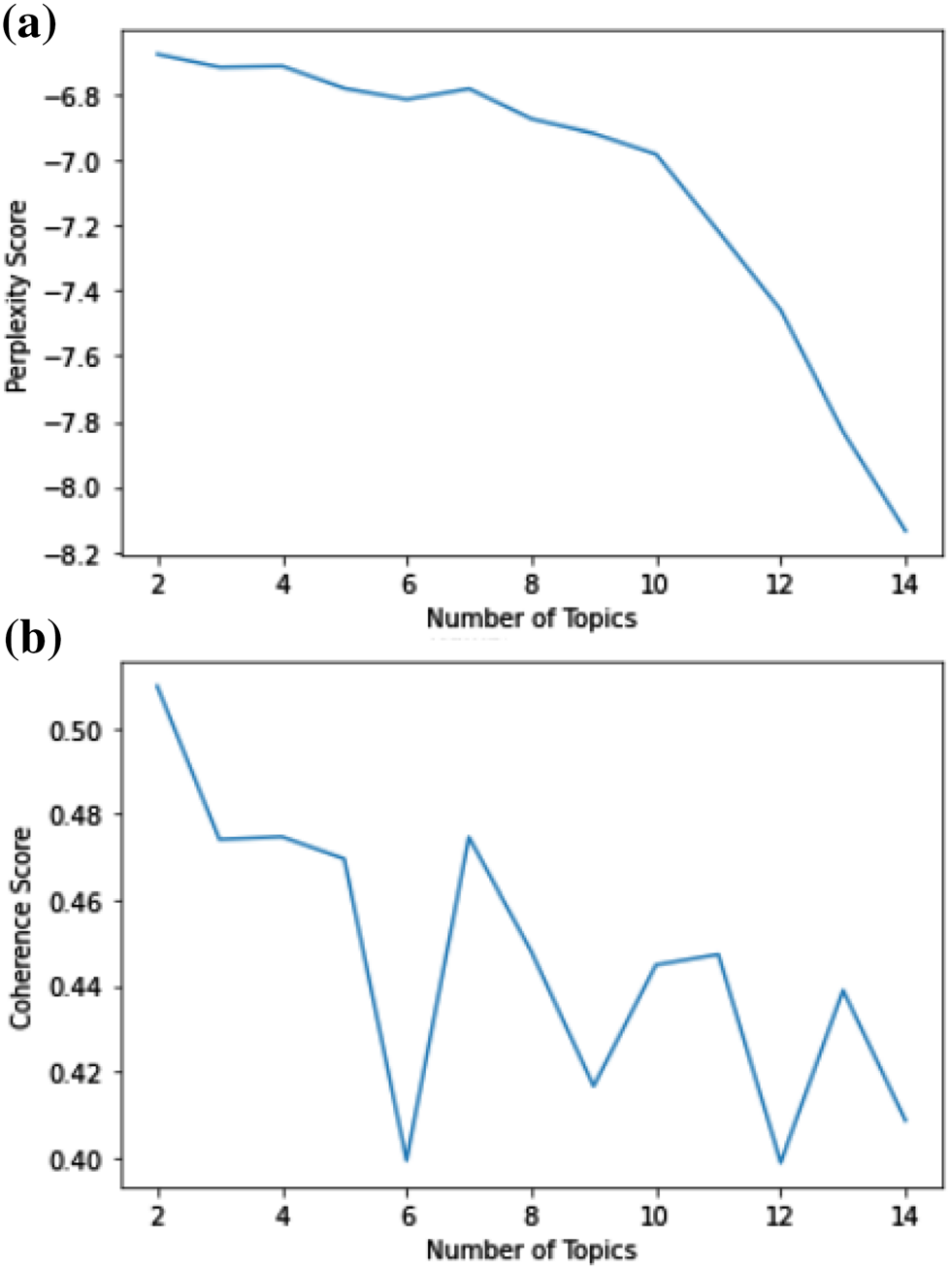
All tweets were evaluated according to the number of different topics: a) Perplexity Score b) Coherence Score

Figure 4 depicts that the best coherence score belongs to two and three number of topics, and later, it declines immediately. As the number of topics increases, peaks and troughs are observed in the coherence graph. By examining the graphics and inter-topic distance maps, and considering the overlapping, the number of appropriate topics according to the graphic was evaluated as three.

**Fig. 5:**
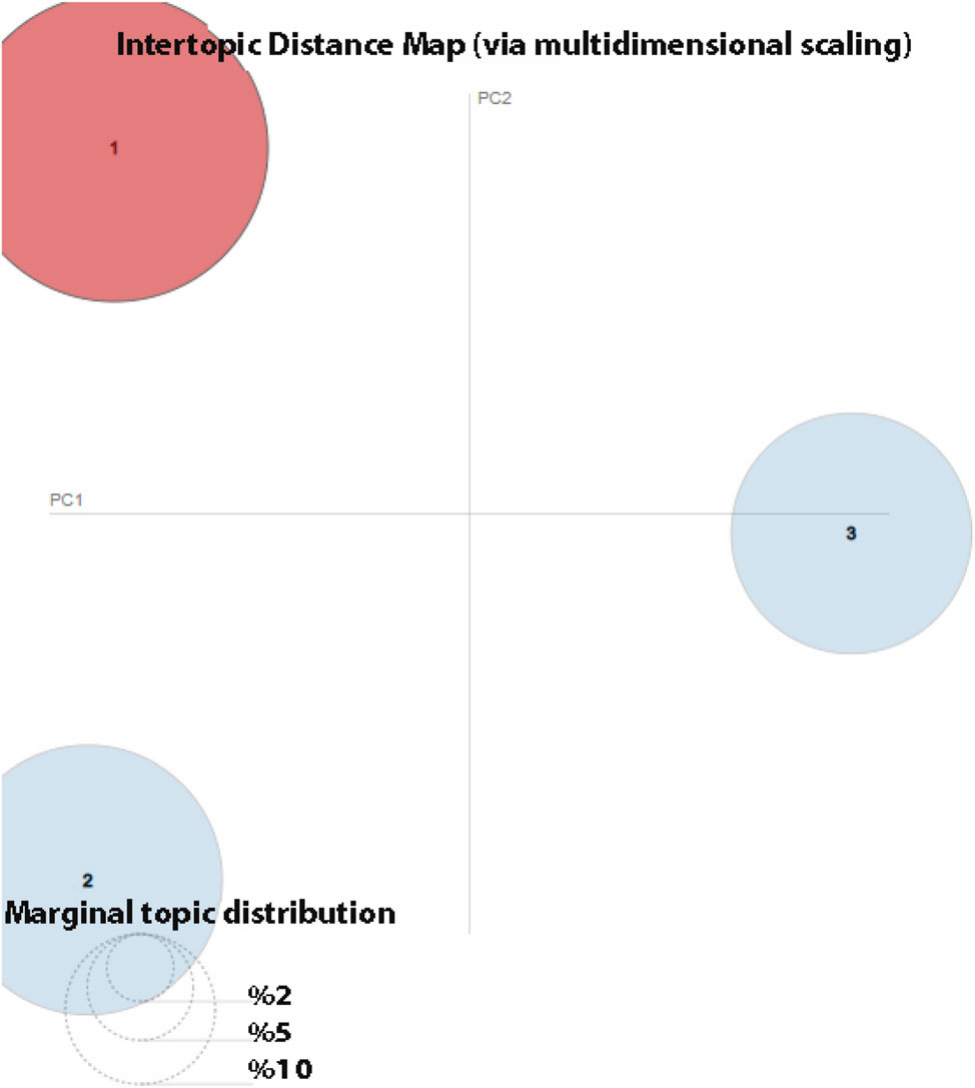
The inter-topic distance map of tweets for sentiment analysis during distance education between 2020-2021

In Fig. 5, the extracted topics for all tweets are shown. Each bubble in this figure represents a topic. The percentage of the number of tweets in the corpus on that topic is related to the size of the bubble. The higher the percentage is, the larger the bubble. Accordingly, topic 1 given in Fig. 5 has the highest percentage. The distance between the bubbles is also related to the similarity of the topics. Bubbles that are far from each other are interpreted as different topics.

**Fig. 6:**
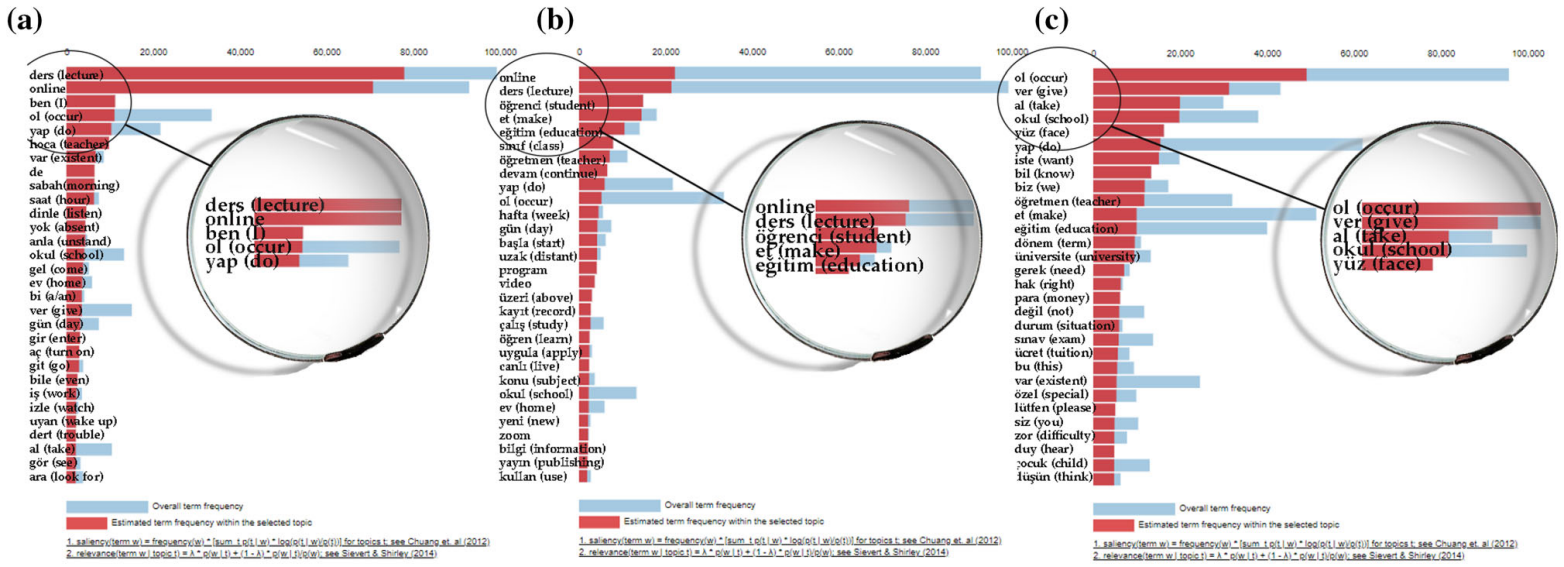
Top-30 most relevant terms for **a** topic 1 **b** topic 2 **c** topic 3

Figure 6 shows the word list for each topic. It expresses its frequency according to the corpus shown in blue in word lists. The bars in red show the number of occurrences of words for the given topic. Accordingly, the words shown with the longest red bar for the given figures are the most used words. It is seen in Fig. 6 that the most used term of topic 1 is the word “ders” (lecture in English). It is seen that the word “online” stands out according to the word list of the second highest percentage on topic 2, and the word “ol” (occurs in English) for topic 3.

**Fig. 7:**
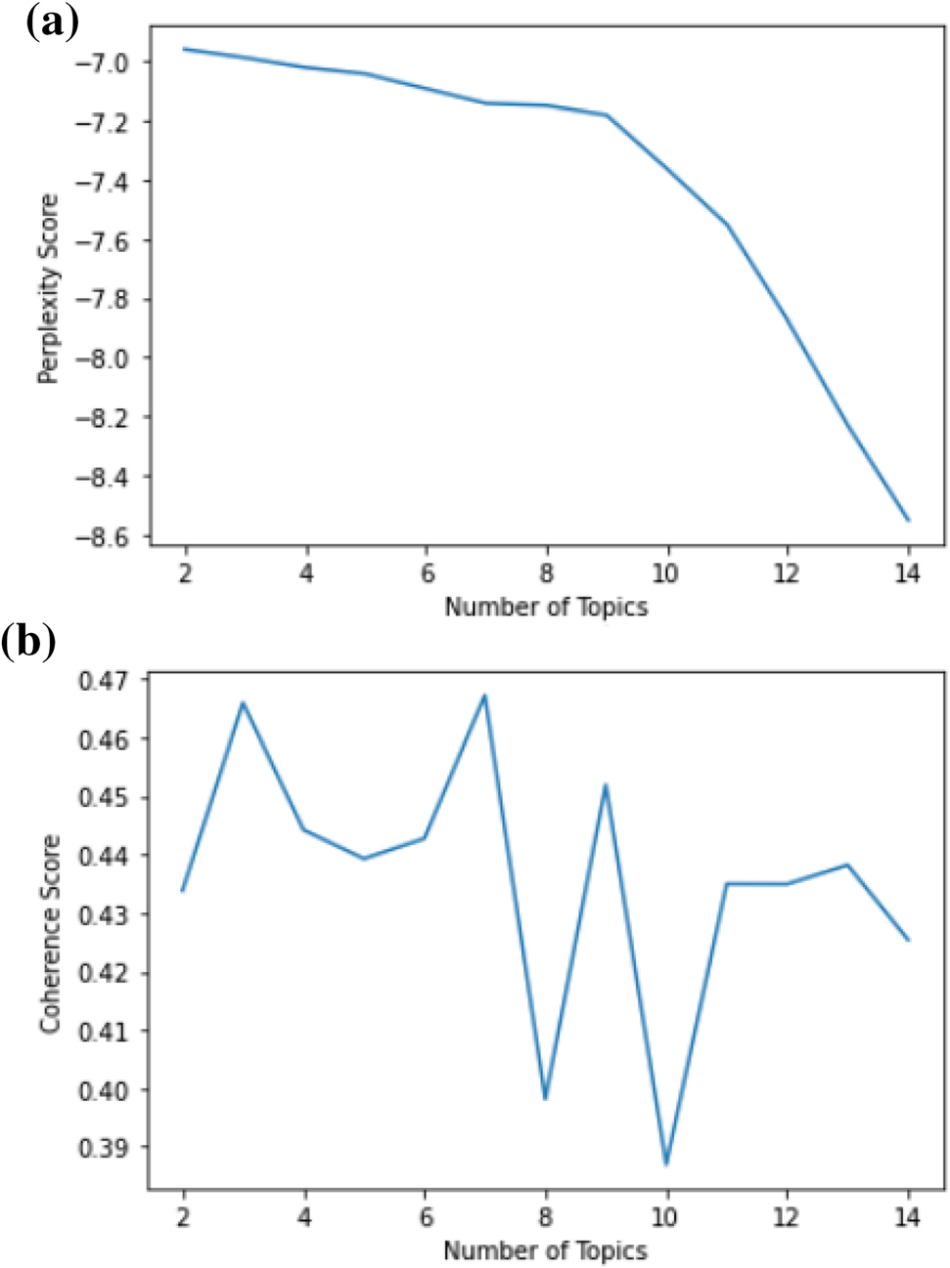
Negative tweets were evaluated according to the number of different topics: a) Perplexity Score b) Coherence Score

Figure 7 depicts that the best coherence scores for negative tweets belong to three and five number of topics. When the topic number was evaluated as 8 and 10, the lowest coherence scores were observed. According to these results, the number of topics with the highest coherence score was determined.

**Fig. 8:**
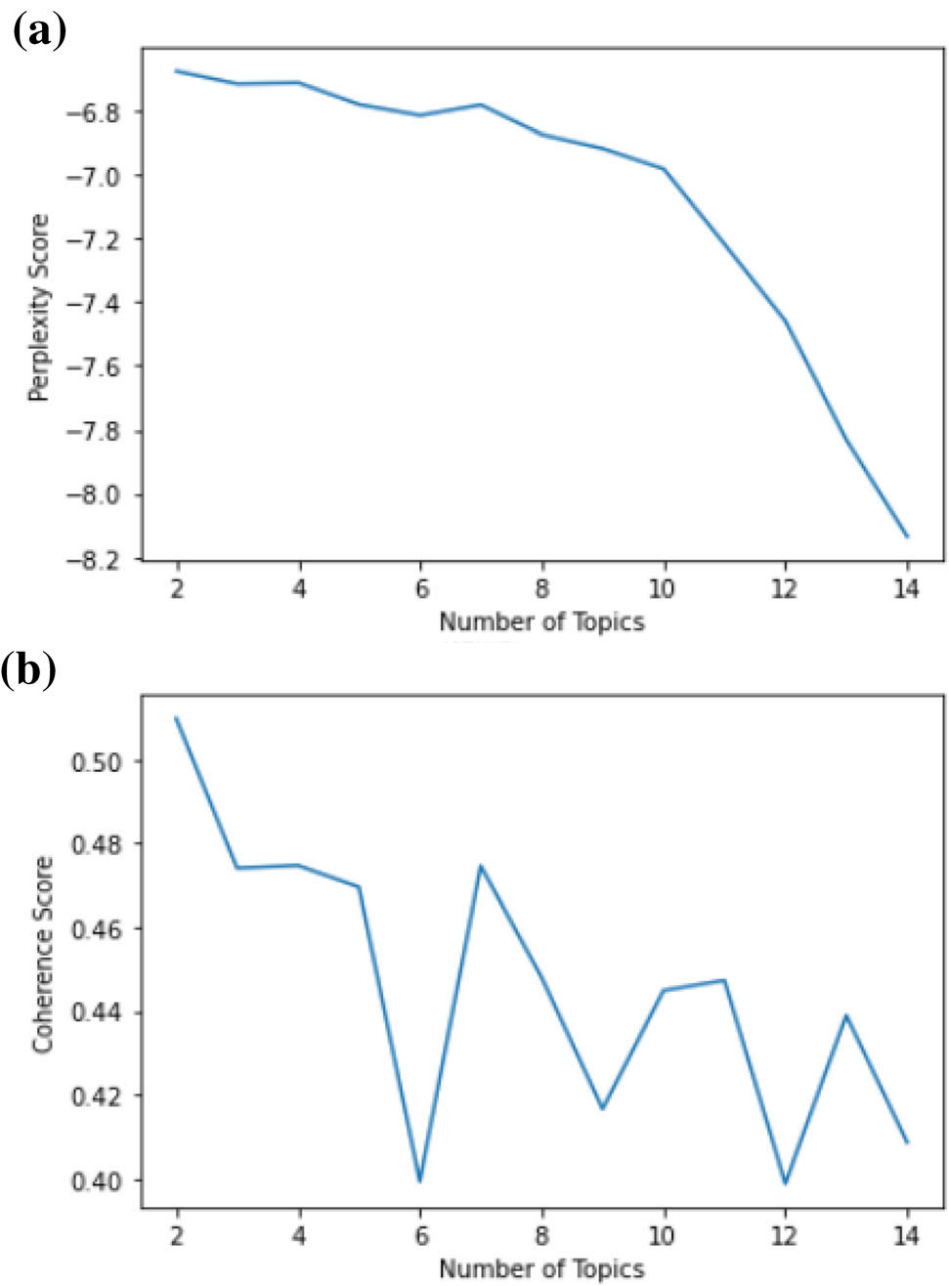
Positive tweets were evaluated according to the number of different topics: a) Perplexity Score b) Coherence Score

When positive tweets are evaluated, the best coherence score is obtained with two number of topics according to Fig. 8. In this graph, the lowest coherence scores were obtained when the number of topics was 6 and 10. The more consistent the subject, the higher the similarity between the words in pairs, so the lowest scores were not taken into consideration in the evaluation of the number of topics, and the number of topics was decided for the high scores.

**Fig. 9:**
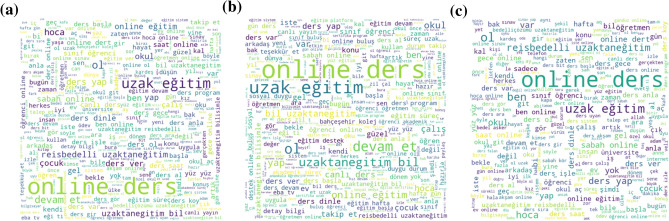
Most frequently used words in **a** all tweets of 2020, **b** negative tweets, **c** positive tweets

A word cloud was also created according to the year of the pre-processed tweets. In word clouds, which contain words formed in different sizes according to their frequencies, size and word importance are directly proportional. As seen in Fig. 9, “online”, “ders” stands out among the words used in all tweets for 2020, as well as for negative and positive tweets.

**Fig. 10:**
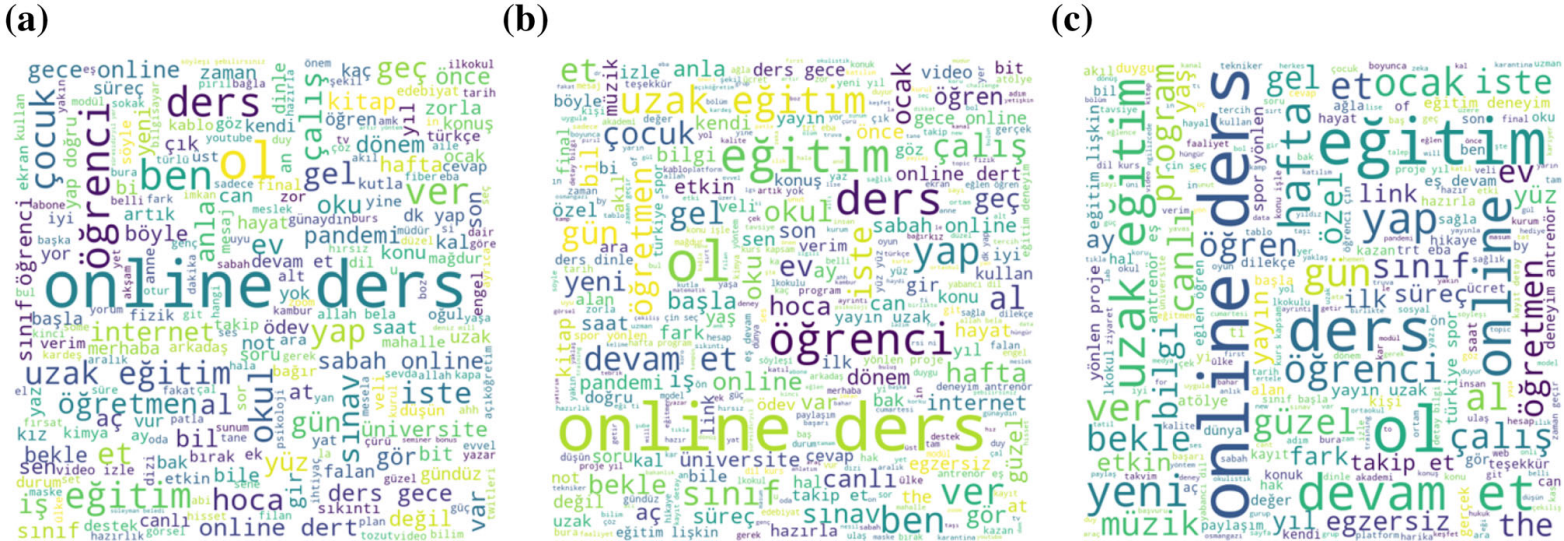
Most frequently used words in **a** all tweets of 2021, **b** negative tweets, **c** positive tweets

As seen in Fig. 10, the words “online”, “ders” stands out again. When both sub-figures in Fig. 9 and 10 are compared, it is seen that words such as “sıkıntı” (distress in English),“dert” (trouble in English),“plan”, “destek” (support in English) are used more frequently in 2021. However, it is seen that words such as “pandemi” (pandemic in English) and “iş” (work in English), which are not seen in the word cloud given in Fig. 9 sub-figure b, are frequently used in tweets classified as negative in 2021 according to sub-figure b in Fig. 10.

**Fig. 11:**
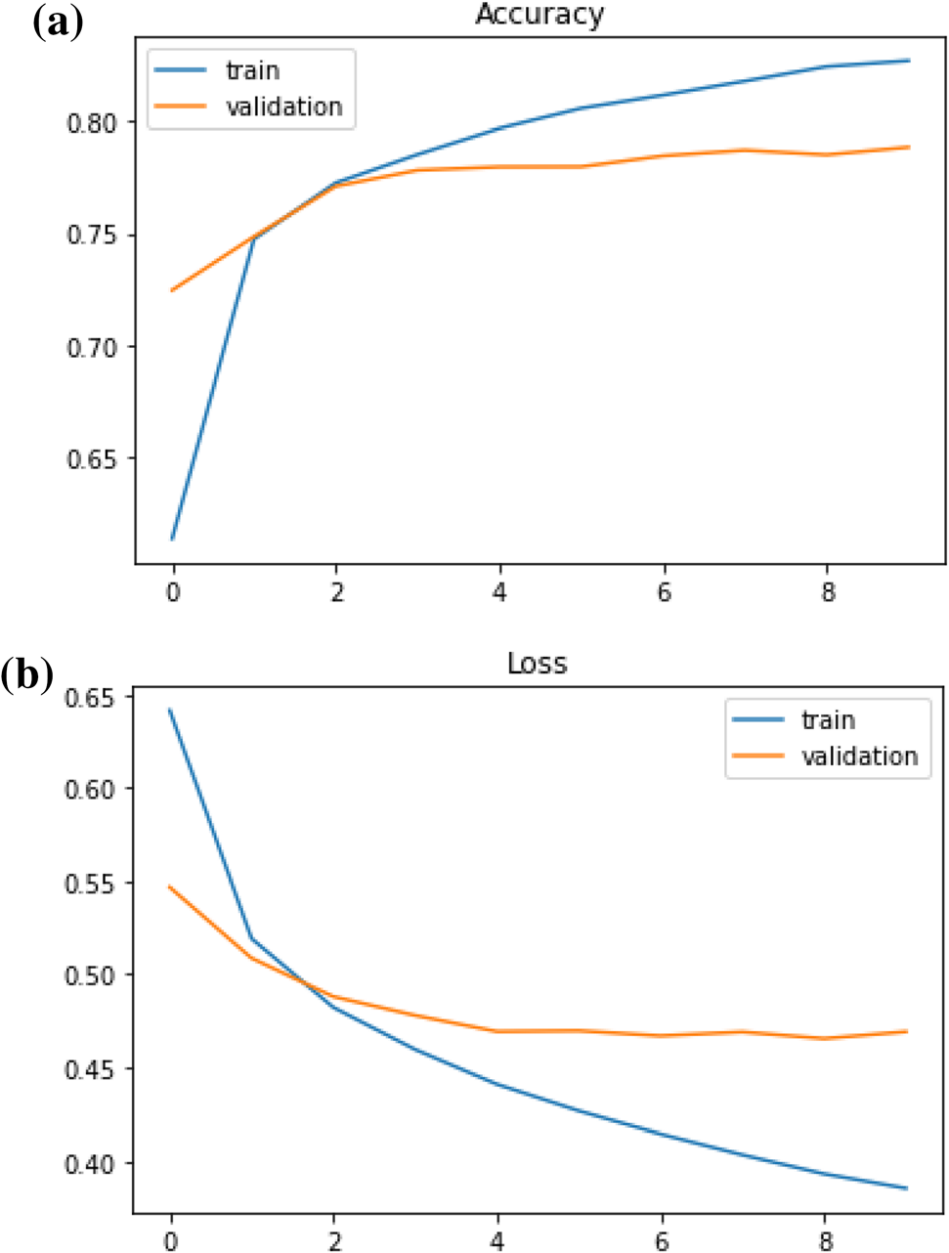
Accuracy and loss trends of the LSTM model

**Fig. 12:**
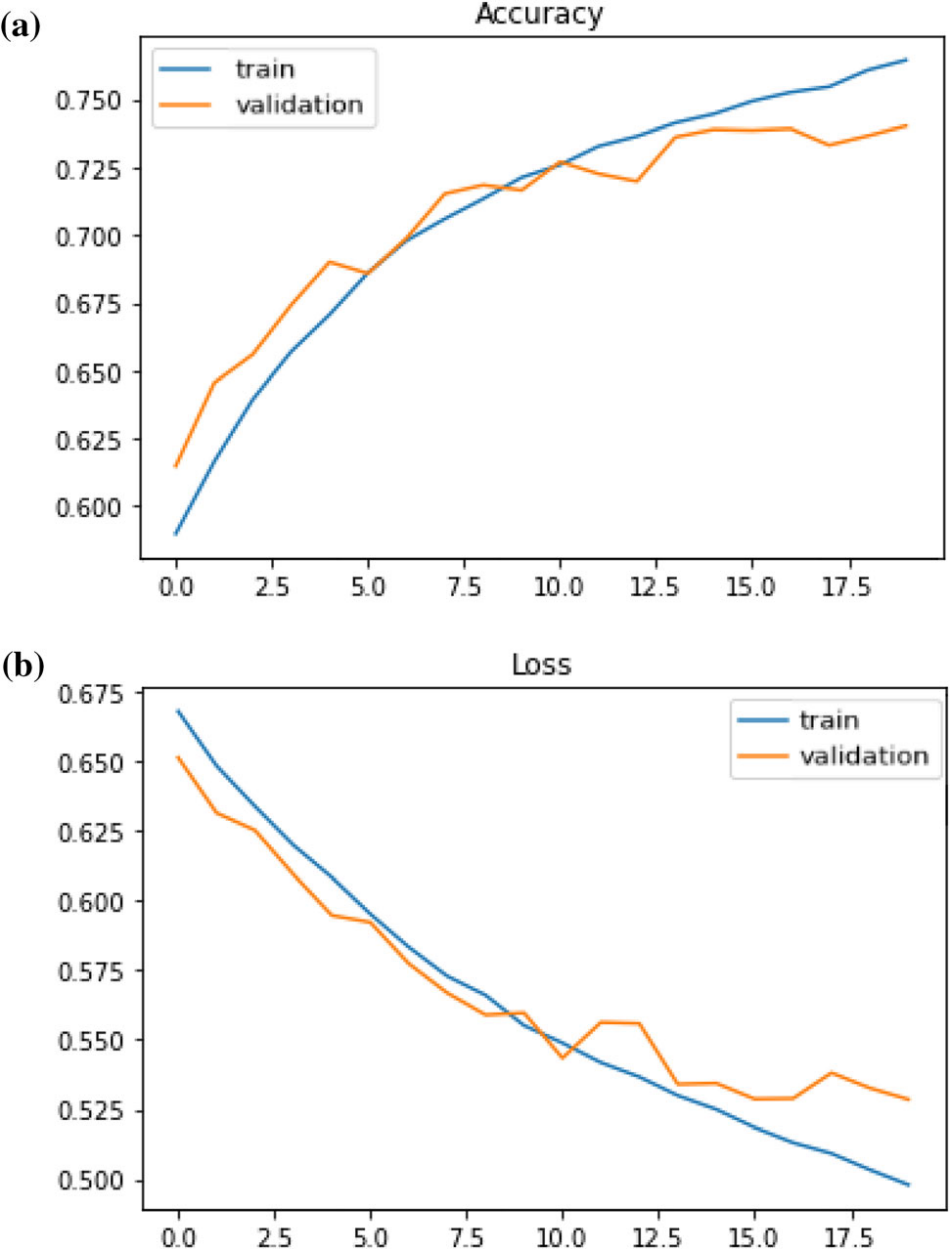
Accuracy and loss trends of the Simple RNN model

After finding the hidden semantic structure with topic modeling, we built NLP models to classify sentiments. We trained the distance education-related Twitter dataset with two deep classifiers namely Long-Short Term Memory (LSTM) and Recurrent Neural Network (RNN). We initiated tuning the models by adding more hidden layers and increasing the number of neurons in the hidden layers to make them more complex, this approach helped us to increase both training and validation accuracies, therefore the models could be learned from and generalized the dataset. However, as the models were getting more complex, the training accuracies continued to increase whilst validation accuracies remained stable, thus we added regularizers and dropout layers to make the accuracy and loss scores of the training and validation sets at equal rates. Also, epoch sizes, optimizers, and learning rates had effects on the learning approach of the models. After some trials of different optimizers, we got the optimal results with Adam in the LSTM model, while SGD gave the most accurate results in the RNN model. We trained the models with several learning rates and got the optimal results with 0.00001 as the learning rate of the LSTM model (Fig. 11 ), and 0.001 as the learning rate of the RNN model (Fig. 12).

**Fig. 13:**
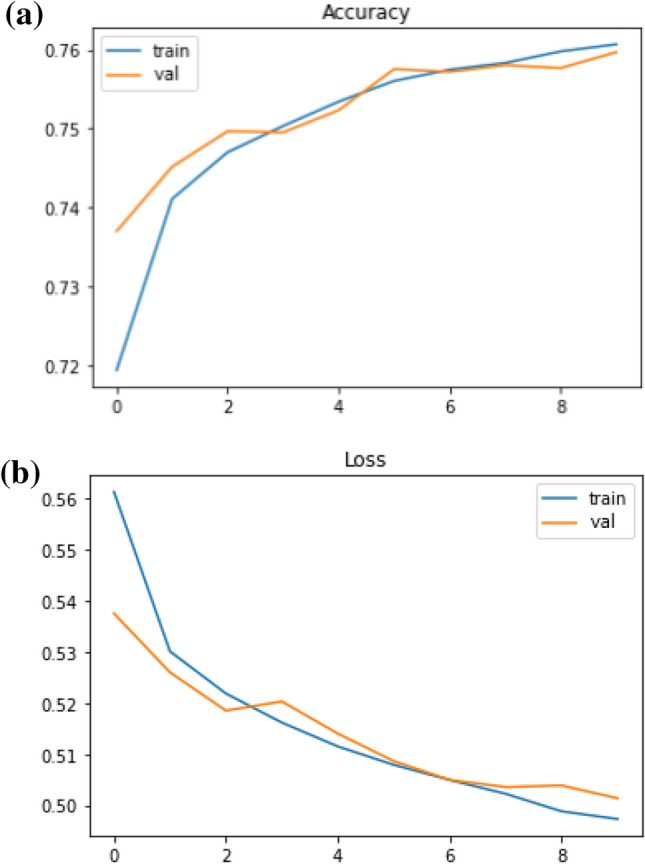
Accuracy and loss trends of the LSTM with Word2Vec model

As it is seen in Fig. 11, LSTM could not have a continuous learning curve after the second epoch. To fix this issue, we fine-tuned the Embedding layer of the LSTM model by changing it with a pre-trained word embedding, which is Word2Vec. (Fig.  13)

When we compare the results (Table 3), we see that the accuracy scores of the algorithms without word embedding are 78% for the LSTM model and 74% for the RNN model. The model trained using word embedding with the same LSTM architecture has a 75.9% accuracy. Although the accuracy of the LSTM without word embedding seems to be higher, it can be said that the LSTM model with Word2Vec has a better learning process when the whole training process is evaluated. This is seen if we compare two LSTM outputs. In the LSTM model after the second epoch, accuracies and losses of the validation curve start to flatten out. However, in the LSTM with the word2vec model, for both training and validation sets, accuracies are increasing and losses are decreasing after each epoch, also, training and validation sets have closer values to each other. Furthermore, the simple RNN model has a consistent learning curve, but when it is compared to the LSTM with word embeddings, LSTM with Word2Vec has better performance.

**Table 3:** Sequential models based sentiment analysis results

Classifier	Val. Acc.	Val. loss	Train Acc.	Train loss
RNN	0.740	0.528	0.764	0.498
LSTM	0.788	0.469	0.827	0.385
LSTM with Word2Vec	0.759	0.501	0.760	0.497

## Conclusion

During the coronavirus pandemic, authorities took some precautions to protect the citizens’ health while meeting societal needs, which led to the closure of traditional face-to-face education institutions and the forced rise of the distance education system. While some countries were more agile in taking measures to improve the quality of education, many countries had a difficult time making this transition due to several problems. This was a very large-scale experiment and unfortunately damaged the resistance and faith of society. This caused psychological issues and caused people to become introverted. Since social media allows people to express themselves in the easiest way, it was critical to make sense of the content here. Therefore, the study is intended to identify the major topics and sentiments about COVID-19 distance education discussed on social media. We also explore the changes in these topics and sentiments awareness to better understand the extensive trend since it is still being discussed if distance education is helpful for learners or not. The first category of contributions focused on finding the general sentiment of individuals. To do so, we gathered the social media posts between 2020 and 2021. Varied text preprocessing techniques, such as tokenization, normalization, stop-word removal, stemming, etc., have been employed to clean the content. Afterward, word embedding approaches have been utilized to assemble neural language models based on word vectors by Word2Vec. Following that, several deep learning-based sentiment models were created, as well as topic modeling using the LDA algorithm. It was observed that word embedding has improved the LSTM-based sentiment model. The highest performance was obtained with the LSTM model using Word2Vec, which has 75.9% validation accuracy. It is observed that in Turkey, according to the tweets that were collected in the given period of time, 54.5 percent of the people have negative feelings about online education. The primary reason for this situation is that, unlike traditional education, education has changed from being a community activity to an individual practice at home, necessitating the need to learn new tools and decreasing social activities by reducing campus access.

## Data Availability

Data is available upon request.

## Footnotes

1 available at https://github.com/twintproject/twint

2 available at https://github.com/ikuceresearch/Turkish-NLP-dataset

3 available at https://github.com/kuzgnlar/models
